# Round Cell Sarcoma: A Challenging Diagnosis

**DOI:** 10.7759/cureus.84225

**Published:** 2025-05-16

**Authors:** Andreia Mandim, Ana Sofia Silva, Rita Moça, Fani Ribeiro, Rubina Silva

**Affiliations:** 1 Internal Medicine, Centro Hospitalar Póvoa de Varzim/Vila do Conde, Póvoa de Varzim, PRT

**Keywords:** aggressive soft tissue neoplasm, diagnostic delay, peritoneal carcinomatosis, tumor lysis syndrome, undifferentiated round cell sarcoma

## Abstract

Sarcomas are malignant tumors of mesenchymal origin that can occur throughout the body. Among these, undifferentiated round cell sarcomas are rare, aggressive tumors, often affecting young adults and presenting diagnostic challenges. We report the case of a 22-year-old female who presented with persistent chest pain and progressive respiratory distress. Despite extensive diagnostic efforts including imaging, thoracentesis, and exploratory laparoscopy, the diagnosis was delayed. Her condition deteriorated rapidly, culminating in sepsis, tumor lysis syndrome, and death. Post-mortem analysis confirmed a diagnosis of undifferentiated round cell sarcoma. This case illustrates the importance of considering rare malignancies in differential diagnoses and highlights the diagnostic and therapeutic challenges posed by these tumors.

## Introduction

Sarcomas comprise a heterogeneous group of malignant tumors that arise from mesenchymal tissues. While they can occur in various anatomical locations, soft tissue sarcomas account for less than 1% of adult malignancies [1]. Undifferentiated round cell sarcomas represent a particularly aggressive and uncommon subgroup, often affecting adolescents and young adults [2,3]. Due to their rarity and the nonspecific nature of initial symptoms, early diagnosis is frequently missed or delayed, which significantly impacts prognosis [4]. The diagnostic process often requires a combination of advanced imaging, histopathological examination, and immunohistochemistry [5]. Here, we present a case that underscores the diagnostic complexity and aggressive clinical progression of round cell sarcoma.

## Case presentation

A 22-year-old woman with a medical history of essential hypertension, dyslipidemia, multinodular thyroid disease, polycystic ovary syndrome, and morbid obesity presented to the emergency department with a three-day history of right-sided chest pain, oppressive in nature, radiating dorsally, worsened by deep inspiration and lying supine. She also reported dyspnea with moderate exertion and episodes of fever, but denied cough, weight loss, night sweats, or recent travel.

Physical examination revealed tachycardia and reduced breath sounds bilaterally at the lung bases. Laboratory tests indicated microcytic anemia, iron deficiency, thrombocytosis, neutrophilic leukocytosis, elevated C-reactive protein and erythrocyte sedimentation rate, subclinical hypothyroidism, and elevated D-dimer levels. A contrast-enhanced thoracic CT revealed bilateral laminar pleural effusion. The patient was admitted for further evaluation.

**Figure 1:**
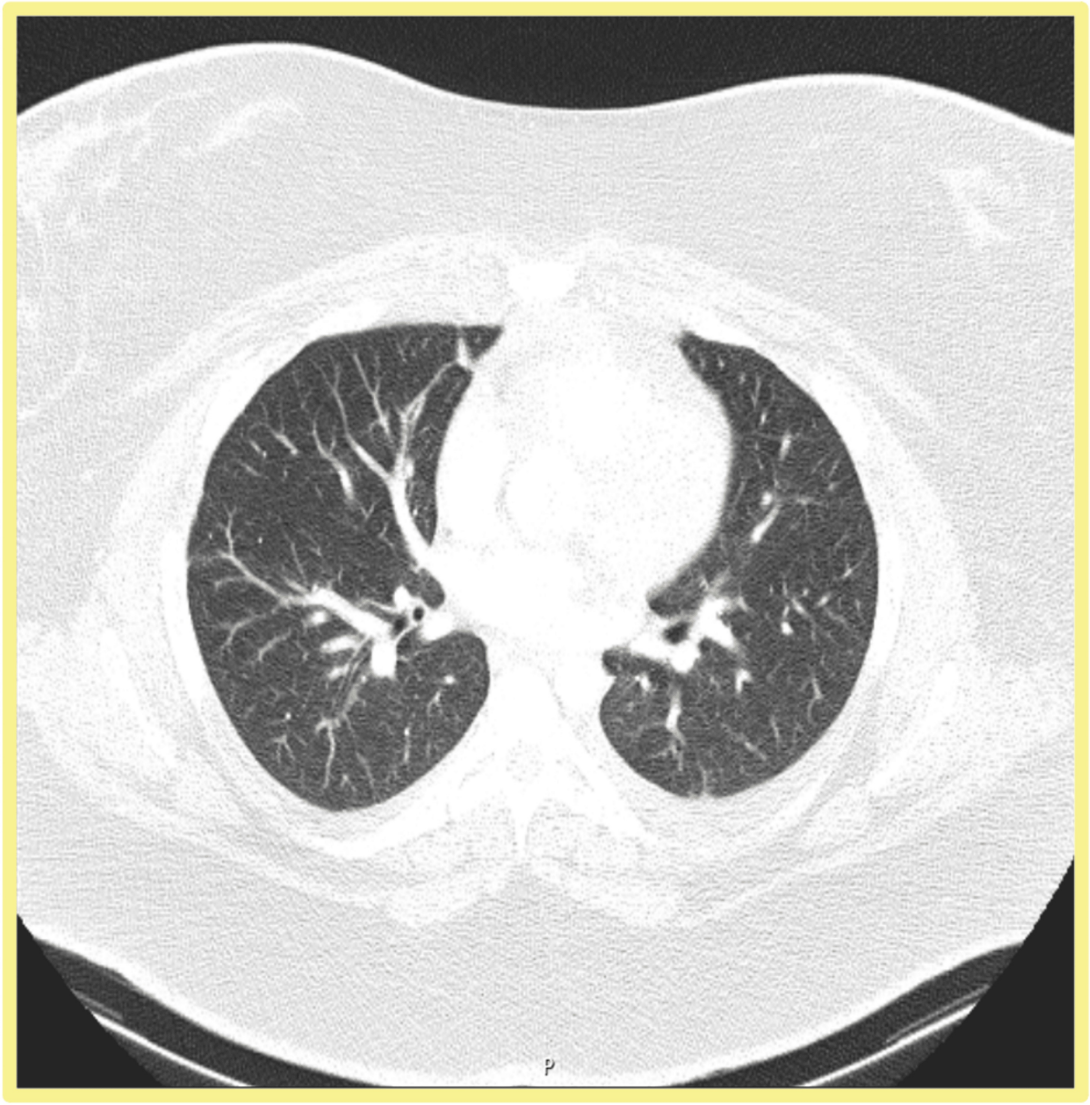
Axial CT of the thorax - lung window. The image demonstrates normal branching of the pulmonary vessels, with no evidence of pulmonary embolism or significant parenchymal abnormalities. No pleural effusion is observed in this slice.

A CT angiography of the chest, abdomen, and pelvis excluded pulmonary embolism but identified pericentimetric mediastinal and abdominal lymphadenopathy, small pleural and pericardial effusions, moderate ascites, and hepatic steatosis (Figure 1). An autoimmune panel was unremarkable. Ultrasound-guided thoracentesis and gynecological evaluation were performed, and peripheral blood immunophenotyping was initiated.

**Figure 2:**
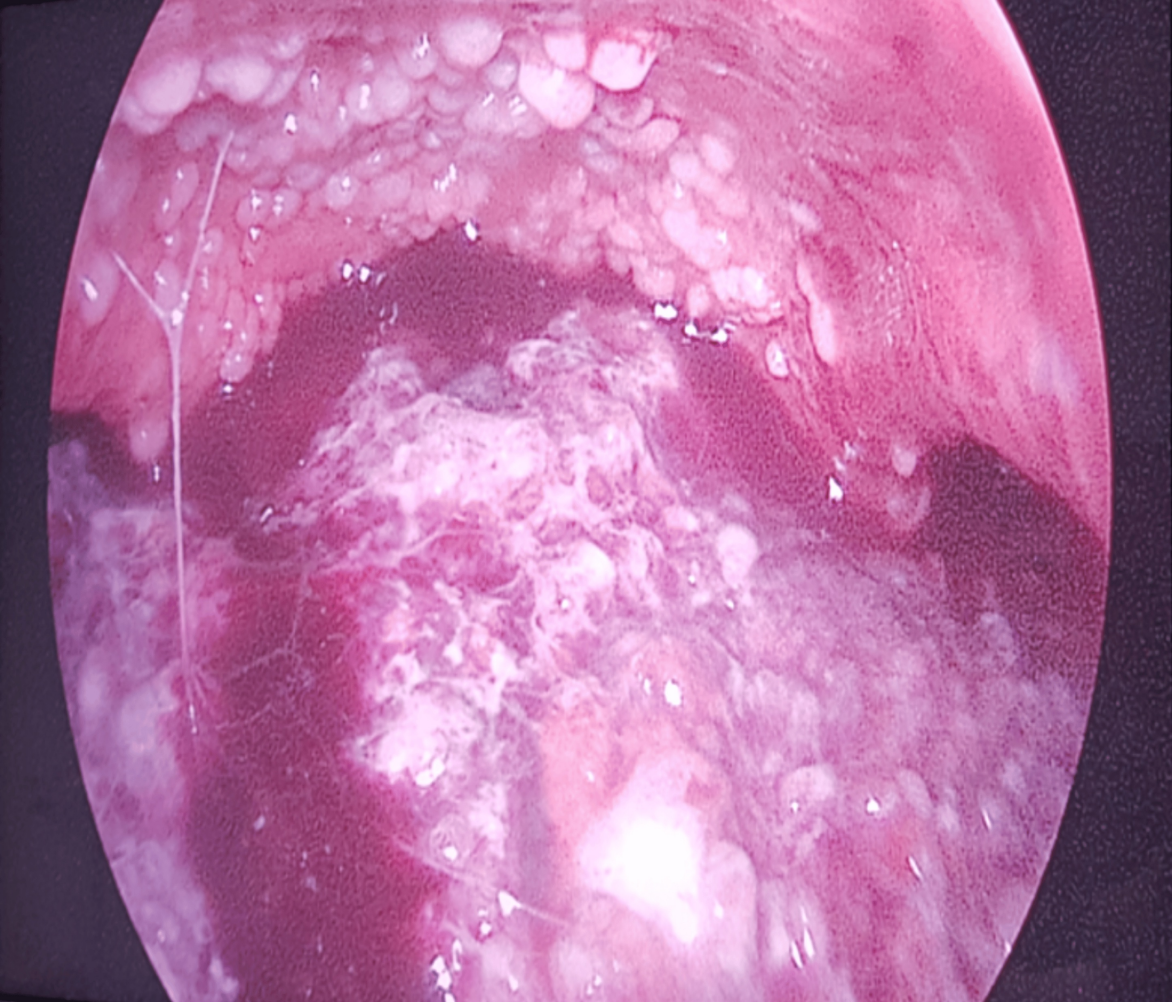
Exploratory laparoscopy. Carcinomatosis implants on the diaphragm and liver surface.

By the third day of hospitalization, the patient developed sepsis of unclear origin and spontaneous tumor lysis syndrome, progressing to severe respiratory failure requiring ICU admission. Exploratory laparoscopy revealed extensive peritoneal carcinomatosis, with friable omental masses and implants on the diaphragm and liver surface (Figure 2). Approximately 6,300 mL of serohematic ascitic fluid was drained.

Flow cytometry of the ascitic fluid and bone marrow aspiration ruled out lymphoma. Small cell carcinoma and desmoplastic small round cell tumor were considered. Despite supportive care, the patient remained critically ill and was not a candidate for tumor-specific therapy. She died on the tenth day of ICU admission. Posthumous cytopathological analysis of the ascitic fluid confirmed a diagnosis of malignant mesenchymal neoplasm composed of undifferentiated round cells with a high mitotic index.

## Discussion

Undifferentiated round cell sarcomas are rare and characterized by high-grade malignancy. They frequently affect young males and often present with nonspecific symptoms such as abdominal pain, distension, or respiratory complaints, depending on tumor location [3,6]. This leads to delayed diagnosis and limits timely intervention.

In our case, despite extensive imaging and invasive diagnostics, a definitive diagnosis was only made post-mortem. Differential diagnoses included lymphoma, gynecologic neoplasms, and other intra-abdominal tumors. The presence of ascites, mediastinal and abdominal lymphadenopathy, and rapid clinical deterioration was suggestive but not conclusive [5,7].

The development of tumor lysis syndrome before confirmed diagnosis was unusual and highlights the aggressive nature of the tumor. Early suspicion and expedited tissue sampling might have improved the diagnostic timeline. However, the patient’s unstable condition precluded more invasive diagnostic procedures or therapeutic interventions.

Histopathology remains the cornerstone for diagnosing undifferentiated sarcomas, often requiring adjunct immunohistochemistry and, increasingly, molecular diagnostics to identify specific translocations or mutations [1,8]. Prognosis depends on tumor grade, size, location, and the patient’s overall condition. In most cases, survival is less than three years from the time of diagnosis [9].

## Conclusions

Undifferentiated round cell sarcomas are highly malignant tumors with poor prognosis and often present with nonspecific clinical features that delay diagnosis. Management is further complicated by the aggressive nature of the disease and rapid clinical decline. This case underscores the importance of early suspicion in atypical presentations and the need for prompt and coordinated diagnostic efforts. Future research should focus on improved molecular characterization and targeted therapies to enhance outcomes in this patient population.
